# Case series: pachychoroid pigment epitheliopathy transformed to polypoidal choroidal vasculopathy after long-term follow-up

**DOI:** 10.1186/s12886-022-02487-8

**Published:** 2022-06-21

**Authors:** Jiyang Tang, Xinyao Han, Ran Tang, Mengyang Li, Zongyi Wang, Mingwei Zhao, Jinfeng Qu

**Affiliations:** 1 Department of Ophthalmology, Peking University People’s Hospital, No.11 Xizhimen South Street, Beijing, 100044 China; 2 Eye Diseases and Optometry Institute, Beijing, China; 3 Beijing Key Laboratory of Diagnosis and Therapy of Retinal and Choroid Diseases, Beijing, China; 4 College of Optometry, Peking University Health Science Center, Beijing, China

**Keywords:** Pachychoroid, Pachychoroid pigment epitheliopathy, Polypoidal choroidal vasculopathy, Pachychoroid spectrum diseases

## Abstract

**Background:**

Pachychoroid pigment epitheliopathy (PPE), a retinal disorder that falls into the pachychoroid spectrum, is characterized by retinal pigment epithelium changes in pachychoroid eyes without existing or previous subretinal fluid or soft drusen. Previous reports have indicated that PPE may share some pathophysiologic component with other pachychoroid spectrum diseases and could transform into central serous chorioretinopathy (CSC) during follow-up. CSC transformation to PNV and PCV has also been reported, but PPE transformation to PCV has not been reported.

**Case presentation:**

Seven eyes of seven patients (four male three female, aged 62.7 ± 8.4 years) who presented with PPE at baseline transformed to PCV during follow-up. All study eyes had baseline contralateral eye diagnoses of PCV. All PPE eyes reported no symptoms at baseline and were followed up regularly for the treatment of their contralateral eyes. All PPE presented as pigment epithelium detachment (PED) at baseline. The mean central macular thickness (CMT) was 217.6 ± 14.6 µm, the mean subfoveal choroidal thickness (SFCT) was 354.9 ± 94.9 µm, and the mean sub-PPE choroidal thickness was 332.3 ± 84.6 µm. The mean PPE width and height were 1326.4 ± 791.4 µm and 58.7 ± 23.6 µm, respectively, at baseline. Disruption of the ellipsoid zone (EZ) was noted in 3 eyes, while choroidal vascular hyperpermeability (CVH) was noted in 5 eyes at baseline. The follow-up period was 75.0 ± 41.1 months, and the mean transformation time was 49.6 ± 24.8 months. All study eyes received no intervention before transformation.

**Conclusions:**

PPE could transform to PCV after a long follow-up period. Regular follow-ups for a long time should be recommended for patients with PPE.

**Supplementary Information:**

The online version contains supplementary material available at 10.1186/s12886-022-02487-8.

## Background

Pachychoroid pigment epitheliopathy (PPE), first coined by Warrow et al. in 2013, is a set of retinal disorders characterized by retinal pigment epithelium changes in pachychoroid eyes without existing or previous subretinal fluid (SRF) or soft drusen [1]. PPE falls into the pachychoroid spectrum of diseases, a set of disorders associated with choroid thickening, which include central serous chorioretinopathy (CSC), pachychoroid neovasculopathy (PNV), polypoidal choroidal vasculopathy (PCV) or aneurysmal type 1 neovascularization (AT1), focal choroidal excavation (FCE) and peripapillary pachychoroid syndrome (PPS) [2]. Pachychoroid is considered when, in the presence of pachyvessels, subfoveal choroidal thickness (SFCT) exceeds 300 µm or an extrafoveal focus has increased choroidal thickness exceeding SFCT by more than 50 µm [2]. PPE, sometimes considered a “forme fruste” of CSC, has been reported to progress to CSC after long-term follow-up or intravitreal steroid administration [3–5]. There have also been reports associating CSC with PNV and PCV [6]. From those reports, there has been speculation that PPE, CSC, PNV and PCV may represent “a continuous disease process triggered by continuity of choroidal malfunction” [7]. However, to date, there have been few studies on the long-term follow-up of PPE, and there have been no reports of PPE progressing to PCV. Here, we report seven cases of PPE transforming to PCV after long-term follow-up.

Pachychoroid was diagnosed when SFCT was more than 300 μm or when extrafoveal focus choroidal thickness exceeded SFCT by at least 50 μm [2, 3, 8, 9]. PPE was diagnosed if there were RPE changes accompanied with pachychoroid and without signs of past or current SRF and soft drusen [1, 2]. The diagnosis of CSC was based on the presence or a history of focal or diffuse leakage on fundus fluorescein angiography (FFA) with SRF confirmed on optical coherence tomography (OCT), signs of gravitational tracts, zonal areas of hyperautofluorescence, or geographic areas of speckled hyperautofluorescence without drusen or choroidal neovascular membrane (CNV). The diagnosis of PNV was based on the presence of pachychoroid with type I CNV confirmed with indocyanine green angiography (ICGA) and/or OCT angiography (OCTA) [10, 11]. The diagnosis of PCV was based on the presence of polypoidal lesions confirmed with ICGA or OCT features such as sharp peaked PED and multilobular PED highly indicative of PCV when hyperfluorescent “polyps” could not be visualized [12]. Eyes with previous ocular surgery other than uncomplicated cataract surgery, preexisting ocular diseases (e.g., uveitis, retinal disorders, optic nerve abnormalities), history of CSC and local or systemic usage of steroids were excluded.

## Case presentation

This was a retrospective case series. Seven eyes of seven patients diagnosed with PPE at baseline transformed to PCV during follow-up were reported. All seven patients initially visited our center for decreased vision or metamorphopsia in their contralateral eyes, and all received a diagnosis of PCV in their contralateral eyes. Due to the latent nature of PPE, none of the patients presented any symptoms in their PPE eyes, and the PPE diagnoses were made incidentally. The patients were followed up regularly while receiving treatment for their PCV eyes.

**Fig. 1:**
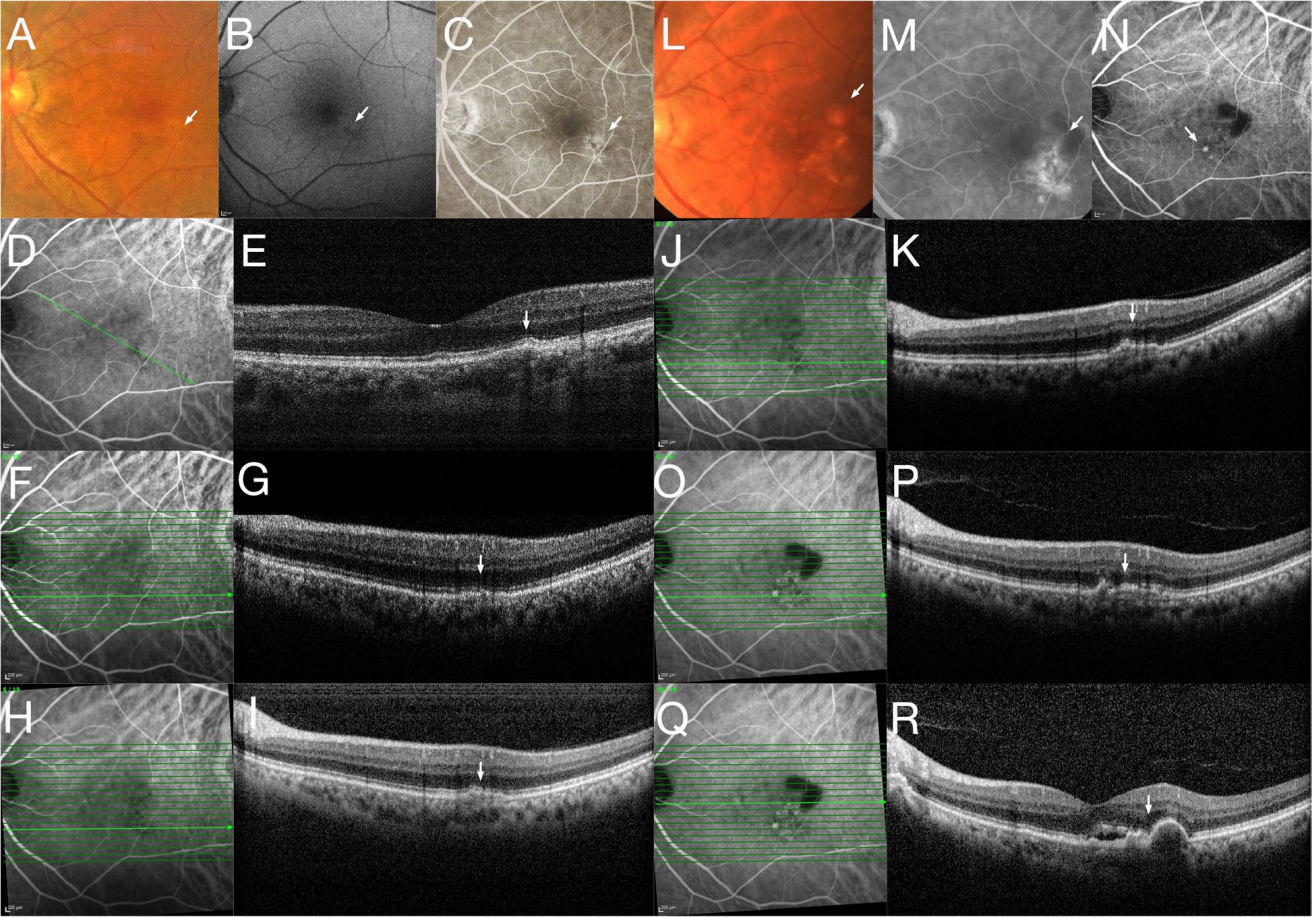
Patient 1 was a 64-year-old male diagnosed with PPE in his left eye. At baseline, fundus examination (**A**) revealed a serous PED in the juxtafoveal region, which appeared on FAF (**B**) as a hypoautofluorescent lesion and a hyperfluorescent lesion (staining) on FFA (**C**). ICGA revealed an area of hypofluorescence (**D**) corresponding to a PED as shown on OCT B-scan (**E**). The PPE lesion showed progressive enlargement during the follow-up. **F**-**G, H**-**I, J**-**K** each showed ICGA images and corresponding OCT B-scans at 45 months, 52 months and 68 months, revealing slow but progressive enlargement of the PPE lesion. Eighty-one months after the initial PPE diagnosis, the patient developed metamorphopsia in his left eye. At 81 months, fundus examination (**L**) revealed a reddish-orange lesion in the juxtafoveal area and serous PED. FFA (**M**) and ICGA (**N**, **O** and **Q**) revealed a hyperfluorescent “polyp” (indicated with white arrow) with an adjacent area of hypoflurescence corresponding to the PED. Corresponding OCT B-scans revealed subretinal fluid (**P** and **R**) and newly developed PED adjacent to the PPE lesion

**Fig. 2:**
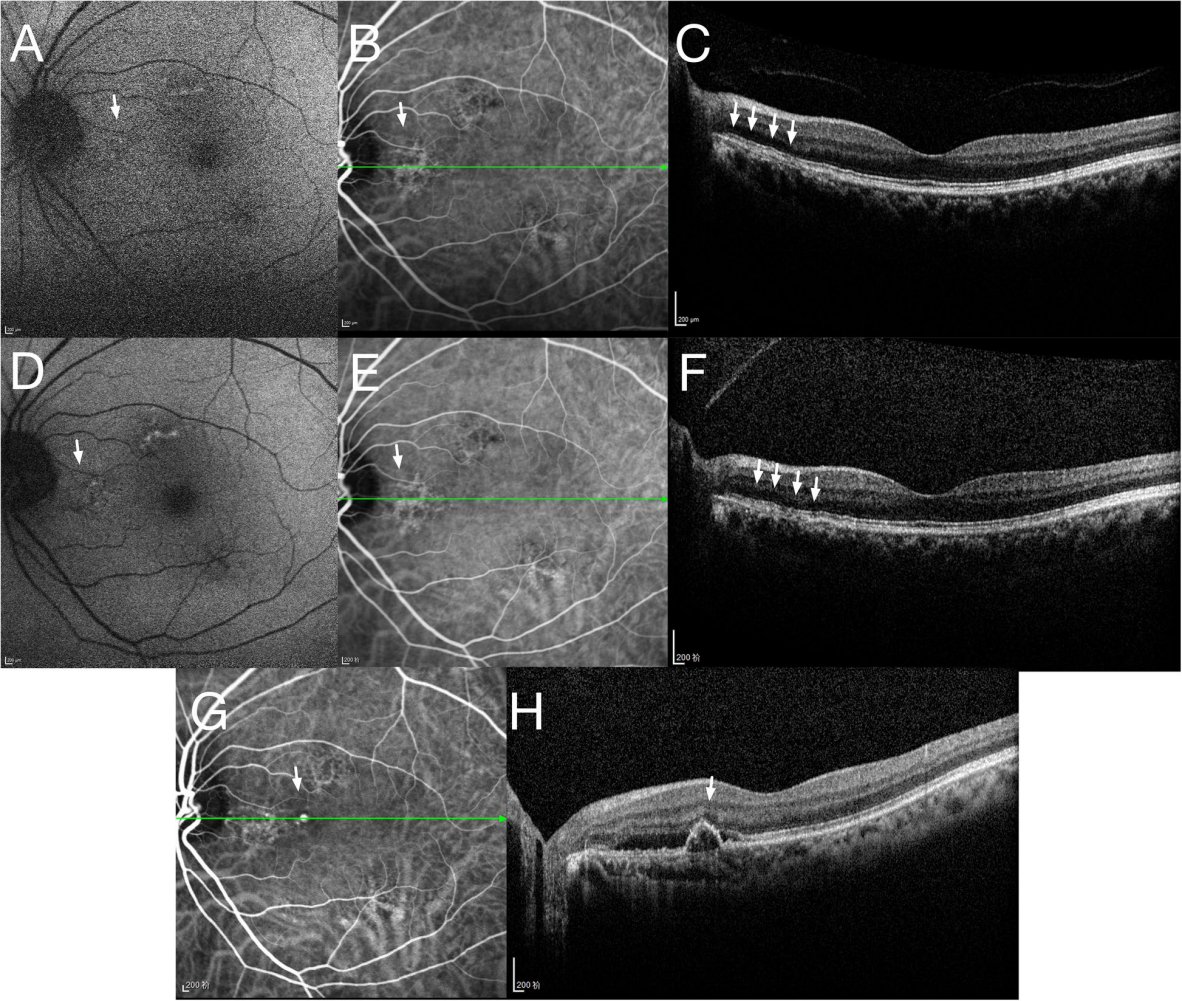
Patient 2 was a 59-year-old female diagnosed with PPE in her left eye. At baseline, FAF (**A**) revealed areas of mottled hyperautofluorescence, while ICGA revealed multiple areas of hyperfluorescence and hypofluorescence (**B**) corresponding to small irregular PED on OCT B-scan (**C**). The lesion showed a stable disease course 12 months after the initial diagnosis. FAF at the 12-month follow-up (**D**), ICGA at the 12-month follow-up (**E**) and OCT B-scan at the 12-month follow-up (**F**) all revealed similar clinical findings with baseline. At 48 months, the patient developed metamorphopsia in her left eye. ICGA revealed a hyperfluorescent “polyp” (**G**, indicated by white arrow) and enlarged PED with subretinal fluid on OCT B-scan (**H**)

**Fig. 3:**
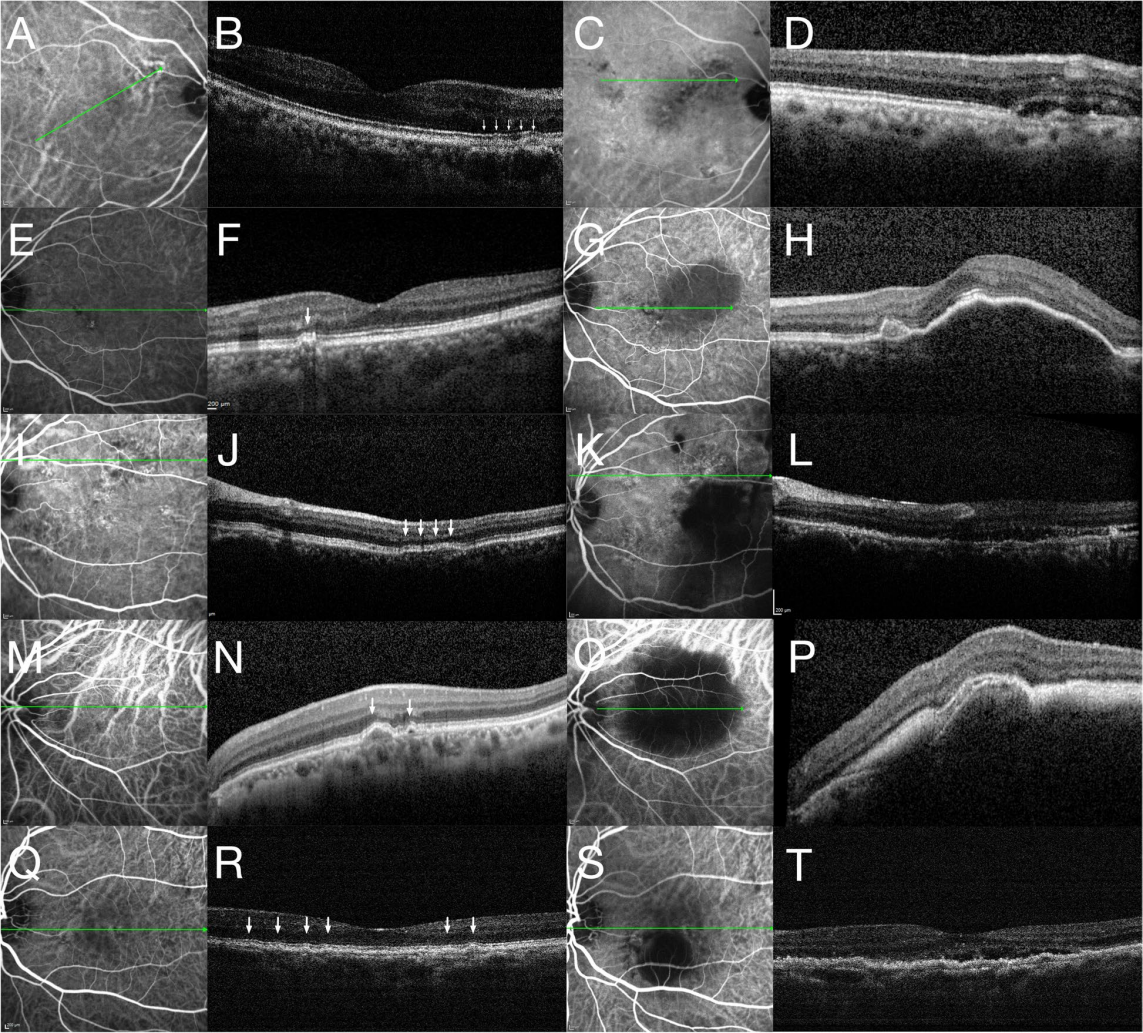
ICGA and OCT images of cases 3–7 at baseline and follow-ups. ICGA images of case 3 at baseline (**A**) revealed pachyvessels, while the corresponding OCT B-scan (**B**) revealed irregular PED at the site of pachyvessels (as indicated by white arrows). After 37 months of follow-up, hyperfluorescent “polyps” were revealed on ICGA images (**C**), and subretinal fluid was noted on OCT images (**D**). ICGA (**E**) and OCT (**F**) images of case 4 at baseline revealed a small PED, while ICGA (**G**) and OCT (**H**) images at 18-month follow-up revealed a hyperfluorescecnt polyp and extensive PED. ICGA (**I**) and OCT (**J**) images of case 5 revealed flat irregular PED at baseline, while ICGA (**K**) and OCT (**L**) images after 84 months revealed subretinal fluid and extensive serous PED. ICGA (**M**) and OCT (**N**) images of case 6 at baseline revealed pachyvessels and small irregular PED, while ICGA (**O**) and OCT (**P**) images after 48 months revealed extensive subretinal hemorrhage. ICGA (**Q**) and OCT (**R**) images of case 7 at baseline revealed small irregular PEDs, while ICGA (**S**) and OCT (**T**) images after 31 months revealed a large serous PED and a “polyp” at the margin of the PED, flat irregular PED and subretinal fluid

All patients were Asian, with four males and three females. The mean age at initial PPE diagnosis was 62.7 ± 8.4 years (median 64 years, range 51–74 years). The patients were followed up for 75.0 ± 41.1 months (median 77 months, range 31–153 months). The mean follow-up interval was 3.1 ± 1.9 months (median 2.4, range 1.5–7.2 months). On their OCT images, all PPE lesions presented with pigment epithelial detachment (PED) with choroid thickening, while retinal pigment epithelium (RPE) migration or irregularity was not noted in these eyes. The mean central macular thickness (CMT) at baseline was 217.6 ± 14.6 µm (median 222 µm, range 203–232 µm), the mean SFCT was 354.9 ± 94.9 µm (median 372 µm, range 230–478 µm), and the mean sub-PPE choroidal thickness was 332.3 ± 84.6 µm (median 309 µm, range 270–507 µm). SFCT of five out of seven contralateral PCV eyes was available. Due to extensive PED and subretinal hemorrhage, the inner surface of the sclera could not be visualized, and the SFCT of the contralateral eyes of case 1 and case 4 could not be measured. The mean SFCT of the contralateral PCV eyes at baseline was 276.2 ± 87.2 µm (median 253, range 190–407 µm). The mean PPE width was 1326.4 ± 791.4 µm (median 1560 µm, range 362–2380 µm). The mean PPE height was 58.7 ± 23.6 µm (median 44 µm, range 36–94 µm). Disruption of the ellipsoid zone (EZ) on optical coherence tomography (OCT) B-scan was noted in three eyes. The PEDs appeared as hyperfluorescent lesions without leakage (staining) on FFA and hypofluorescent lesions and no leakage on indocyanine green angiography (ICGA) (Fig. 1C). No evidence of active CNV, such as subretinal or intraretinal fluid, was noted in any patients on FA or ICGA. Choroidal vascular hyperpermeability (CVH) on ICGA was noted in five eyes. The mean transformation time to PCV was 49.6 ± 24.8 months (median 48 months, range 18–84 months). All patients did not complain of sudden decrease in vision or metamorphopsia until the development of PCV, as confirmed by our multimodal imaging findings in their PPE eyes during regular follow-ups for treatment for their contralateral eyes (Figs. 1, 2 and 3). As the disease courses of all patients followed a similar trajectory, only two representative cases are described in detail below. The demographic and clinical characteristics of all patients at baseline and follow-up information are listed in Table 1. The treatment received and the clinical features of all study eyes at the last follow-up are shown in Supplemental Table 1.

**Table 1:** Demographic and clinical characteristics of all patients at baseline

Patient, n	Sex	Age	BCVA Snellen /LogMAR	CMT (µm)	SFCT^a^ (µm)	Sub-PPE choroidal thickness^b^ (µm)	PPE width^c^ (µm)	PPE height^d^ (µm)	Follow-up time (months)	Time to PCV (months)	Disruption of EZ	CVH	Co-existing medical conditions
1	M	64	0.80/0.10	223	230	309	382	42	153	81	No	No	HTN
2	F	59	1.00/0.00	213	372	270	1560	44	62	48	No	Yes	OA
3	M	74	0.40/0.40	207	380	251	888	36	83	37	No	Yes	DM
4	F	55	1.00/0.00	232	478	507	362	65	31	18	Yes	Yes	DM, HTN
5	F	51	0.63/0.20	223	446	307	1763	43	86	84	Yes	Yes	HTN
6	M	64	1.00/0.00	222	237	358	1950	87	77	48	Yes	No	None
7	M	72	0.63/0.20	203	341	324	2380	94	33	31	No	Yes	None

*BCVA* Best corrected visual acuity, *LogMAR* Logarithm of the minimum angle of resolution, *CMT* Central macular thickness, *SFCT* Subfoveal choroidal thickness, *PPE* Pachychoroid pigment epitheliopathy, *PED* Pigment epithelium detachment, *PCV* Polypoidal choroidal vasculopathy, *EZ* Ellipsoid zone, *CVH* choroidal vascular hyperpermeability, *HTN* Hypertension, *OA* Osteoarthritis, *DM* Diabetes mellitus

^a^Choroidal thickness was measured from the outer portion of the hyperreflective line indicating RPE to the inner surface of sclera on OCT. SFCT was measured at the center of the macula

^b^Sub-PPE choroidal thickness was measured at the center of the PPE lesion on OCT

^c^PPE width was measured from the outer portions of the widest points of the PPE lesions on OCT. If there was no normal appearing RPE between closely located PPE lesions, these lesions were counted as a single lesion

^d^PPE height was measured from the highest point of PPE lesions to Bruch’s membrane

### Case 1

Case 1 was a 64-year-old male who presented with decreased vision and metamorphopsia in his right eye for a month. He was diagnosed with PCV in his right eye and PPE in his left eye. The patient reported no previous episodes of metamorphopsia in either eye or any previous ocular history. He had medically controlled hypertension and no other systemic disorders. Best corrected visual acuity (BCVA) was found to be 0.25 in the right eye and 0.8 in the left eye. Anterior segment examination was unremarkable. Dilated funduscopic examination showed extensive subretinal fluid and PED at the macula in his right eye and PED in the juxtafoveal region in his left eye (Fig. 1A), which showed fundus autofluorescence (FAF) as a hypoautofluorescent lesion (Fig. 1B). FFA revealed a hyperfluorescent staining lesion without leakage in the juxtafoveal region in the left eye (Fig. 1C). ICGA revealed a small area of hypofluorescence corresponding to the PED lesion (Fig. 1D). An OCT B-scan revealed a small PED as well as focal choroid thickening and the presence of pachyvessels beneath the PED (Fig. 1E). SFCT and sub-PPE choroidal thickness were 230 µm and 309 µm, respectively. The PPE width and height were 382 µm and 42 µm, respectively, at baseline. The patient then received one session of photodynamic therapy (PDT) and multiple anti-vascular endothelial growth factor (VEGF) injections for his fellow eye and was followed up regularly. Over the course of the regular follow-up, progressive enlargement of the PED in his left eye was observed (Fig. 1F-K). Eighty-one months after the initial PPE diagnosis, the patient complained of metamorphopsia in his left eye. On this clinical visit, dilated fundus examination revealed a reddish-orange lesion in the juxtafoveal area and PED in the left eye (Fig. 1L) that appeared as a hyperfluorescent lesion with leakage on FFA (Fig. 1M). OCT B-scans revealed newly developed subretinal fluid and serous PEDs (Fig. 1P and 1R), while ICGA revealed hyperfluorescent polypoidal lesions next to the hypofluorescent PED lesions (Fig. 1N, O and Q) but no CVH in the left eye.

### Case 2

Case 2 was a 59-year-old female who presented with decreased vision in her right eye. She was diagnosed with PCV in her right eye and PPE in her left eye. No previous episodes of metamorphopsia in either eye or any previous ocular history were reported. The patient had osteoarthritis (OA) and no other systemic disorders. The BCVA was found to be 0.05 in the right eye and 1.0 in the left eye. Anterior segment examination was unremarkable. Dilated funduscopic examination showed subretinal hemorrhage at the macula in her right eye and multiple areas of slight hyperpigmented and hypopigmented RPE changes that appeared as mottled hyperautofluorescent areas on FAF (Fig. 2A) in her left eye. An OCT B-scan revealed small flat irregular PEDs in her left eye (Fig. 2C). The SFCT and sub-PPE choroidal thicknesses were 372 µm and 270 µm, respectively. The PPE width and height were 1560 µm and 44 µm, respectively, at baseline. ICGA revealed areas of hyperfluorescence and hypofluorescence without leakage corresponding to the PPE lesion (Fig. 2B). The patient then received multiple anti-VEGF injections for her contralateral eye and was followed up. The PPE lesion in her left eye remained stable (Fig. 2D-F) until 48 months after the initial PPE diagnosis, when the patient complained of metamorphopsia in her left eye. At this clinical visit, her OCT B-scan images revealed “thumb-like protrusion” and subretinal fluid as well as the preexisting small irregular PED (Fig. 2H), while ICGA revealed a hyperfluorescent “polyp” at the place of the original PPE lesion in her left eye (Fig. 2G).

## Discussion and conclusions

Pachychoroid spectrum disease is a relatively new concept that has received increasing academic and clinical attention in recent years. It is a set of diseases characterized by focal or diffuse choroidal thickening, thinning of the inner choroid and CVH [2]. Among the many disorders included in the pachychoroid spectrum, PPE, CSC, PNV and PCV and their associations have been discussed in detail in the literature.

Due to the asymptomatic nature of PPE and its underestimated prevalence in the population, there have been few large-scale studies on its natural disease course. Previously, Saito et al. reported a PPE case that transformed to CSC after 35 months of follow-up [5]. Warrow et al. followed up eight patients with PPE for a mean of 70 months and suggested that PPE might progress to CSC, but these eyes tended to remain stable and did not progress [1]. Karacorlu retrospectively reviewed 46 PPE patients with at least 3 years of follow-up and found 48 eyes (82.6%) with a stable clinical course, while 8 eyes (17.4%) developed CSC, but none developed PCV [3]. Ersoz et al. reported a case of a PPE eye developing CSC after five consecutive intravitreal administrations of dexamethasone [4]. Another study by Ersoz et al. showed that the outer nuclear layer (ONL) in PPE eyes was thinner than uncomplicated pachychoroid and healthy eyes, indicating that the degenerative process of pachychoroid spectrum diseases may begin with RPE alterations before the onset of subretinal fluid [13], lending credence to the theory that PPE could be part of the same disease process that eventually leads to PCV. Although CSC have also been associated with PCV [6], there have been no previous reports of PPE transforming to PCV. In this case series, we reported seven cases of PPE developing into PCV after long-term follow-up periods. The average transforming time was 49.6 ± 34.4 months (median 48 months, range 18–84 months).

Karacorlu et al. classified PPE into four types: RPE elevation with microbreak appearance, PED, RPE thickening and hyperreflective RPE spikes [3]. It is interesting to note that in our case series, most PPE lesions initially presented with flat irregular PED (FIPED) (Fig. 3, Supplemental Figs. 1, 2, 3, 4 and 5). Previous studies have shown that FIPED in the context of CSC was associated with type 1 neovascularization [11], although whether this type of PPE alone is more likely to develop into PCV needs further investigation. Another risk factor for transformation is the diagnosis of their fellow eye. In our report, all patients had PCV in their fellow eye. A study by Sakurada et al. showed that reduced choriocapillaris flow density, increased choroidal thickness and CVH appeared to colocalize in eyes with PPE, indicating that inner choroidal ischemia was likely to be associated with the pathogenesis of PPE [14]. PPE eyes that transformed to PCV might have more severe thinning of the inner choriocapillaris, which further results in ischemia and disruption of the RPE [15]. Future longitudinal studies of choroidal circulation hemodynamics in patients with PPE are needed to investigate the pathogenic difference between those transformed to PCV and those that did not.

Previous studies on fellow eyes of PCV reported that PCV could develop in 9% to 17% of fellow eyes after long-term follow-up [16, 17]. A few studies have reported on precursor lesions of PCV. Kang et al. reported drusen-like deposits and pigmentary changes in contralateral eyes of PCV [18]; Kim et al. reported fellow eyes of PCV that developed PCV or CNV during follow-up presented more irregular RPE elevation, branching vascular network, thicker choroid, choroidal vascular dilation and CVH [16]; Baek et al. reported RPE and outer retinal abnormalities accompanied by pachyvessel increased risk of neovascularization development for fellow eyes of PCV [17]. These reports were consistent with our reports where RPE abnormalities could be precursor lesions of PCV, although previous studies focused more on contralateral eyes of unilateral PCV, while our case series reviewed the study eyes in the context of pachychoroid spectrum diseases, showing that PPE lesions could progress to PCV after long-term follow-up, providing evidence that PPE could be precursor lesions of PCV and that pachychoroid spectrum diseases could be a continuous disease process.

There are limitations to this case series. As this was a retrospective case series, the follow-up interval was not standardized; therefore, the lesions reported were not observed on a regular basis. Patient history revealed no sudden change in visual acuity or the onset of metamorphopsia, micropsia, or central scotoma before symptoms of active PCV, indicating that there was a low likelihood of subretinal fluid during the follow-up period. However, the patients in this case series did not receive OCTA examination due to lack of knowledge of this spectrum of diseases at the time and the unavailability of OCTA at our center at the time of earlier clinic visits. While all patients in our case series underwent dye angiography to exclude active neovascularization, OCTA has been reported to be more sensitive in the detection of CNV [11]. Previously, Lee et al. reported that latent PNV could remain asymptomatic and without SRF for a long period of time [19]. Therefore, it could not be excluded that the PPE lesions reported in this case series first progressed to latent nonexudative PNV before transforming to PCV. In the same study by Lee et al., they observed that nonexudative PNV were more prone to develop PCV during follow-up, although whether nonexudative PNV had more linkage with PCV than exudative PNV remains to be seen, as treatment received for exudative PNV might affect the natural disease course of PNV [19]. Studies into genetic risk factors for PCV have shown that genetic variations in ARMS2 and complement Factor H gene (CFH) increased the risk of PCV [20]. We did not investigate the genetic variations in our patients, so it could not be excluded that their transformation may be related to undiscovered genetic variation rather than PPE itself. The diagnosis of PCV in their fellow eyes gave these patients a higher chance to be diagnosed with PPE and followed closely, which resulted in detection bias and may give a false picture of PPE transformation. Large-scale prospective studies of PPE are needed to confirm this speculation.

Since most PPE patients are asymptomatic, usually no treatment is given unless they develop CSC, PNV or PCV. However, our cases showed that PPE may transform to vision-threatening PCV in the long run; therefore, we need to consider whether early intervention with PPE will benefit our patients, especially those with PCV in their fellow eyes.

In conclusion, PPE could transform to PCV after a long follow-up period. Our cases lent more credence to the theory that PPE, CSC, PNV and PCV could be a continuous disease process. Long-term regular follow-ups should be recommended for patients with PPE.

## Supplementary Information

**Additional file 1:** **Supplemental Figure 1. **Multimodal imaging of case 3 at baseline and 37-month follow-up. Fundus image at baseline (A) revealed pigmentary changes at the posterior pole. The ICGA image at baseline (B) revealed dilated choroidal vessels. An OCT-B scan at baseline (C) revealed flat irregular PED accompanied by pachyvessels corresponding to the location of pigmentary changes on fundus images. The fundus image at the 37-month follow-up (D) revealed subretinal fluid at the posterior pole. The ICGA image at the 37-month follow-up (E) revealed a hypofluorescent lesion (corresponding to the area with subretinal fluid) and hyperfluorescent “polyps”. An OCT B-scan at the 37-month follow-up (F) showed subretinal fluid and flat irregular PED.

**Additional file 2:** **Supplemental Figure 2. **Multimodal imaging of case 4 at baseline and 18-month follow-up. The fundus image at baseline (A) revealed two areas with pigmentary changes. Fundus autofluorescence (B) revealed a hyperautofluorescent lesion (indicated with white arrow) and a hypoautofluorescent lesion (indicated with green arrow). FFA at baseline (C) revealed two hyperfluorescent lesions; the white arrow indicates the area of staining hyperfluorescence, while the green arrow indicates the area of the window defect. (D) ICGA at baseline revealed a hyperfluorescent lesion corresponding to a PED on OCT B-scan (E). The fundus image at the 18-month follow-up (F) revealed extensive subretinal hemorrhage. ICGA images (G) at the 18-month follow-up revealed an area of hypofluorescence corresponding to the area with subretinal hemorrhage and a hyperfluorescent “polyp” (indicated with white arrow).

**Additional file 3:** **Supplemental Figure 3. **Multimodal imaging of case 5 at baseline and follow-up. The infrared reflectance (IR) image at baseline (A) revealed mottled reflectivity at the posterior pole. ICGA images (B) revealed areas of hyper- and hypo-fluorescence corresponding to serous PED on OCT B-scan (C). IR image (D), ICGA image (E) and OCT B-scan at 61-month follow-up revealed a stable clinical course. The IR image at the 84-month follow-up (G) revealed a large area of decreased reflectivity corresponding to extensive subretinal hemorrhage. FA at the 84-month follow-up (J) revealed mottled hyperfluorescence adjacent to an extensive area of hypofluorescence (masking). ICGA images at the 84-month follow-up (H and K) revealed a branching vascular network adjacent to an extensive area of hypofluorescence caused by masking. An OCT B-scan revealed subretinal fluid with flat irregular PED (I) and hemorrhagic PED (L).

**Additional file 4:** **Supplemental Figure 4. **Multimodal imaging of case 6 at baseline and 48-month follow-up. FAF at baseline (A) revealed an area of hyperautofluorescence corresponding to hypofluorescence (masking) on ICGA images (B). An OCT B-scan revealed irregular PED with hyperreflective material. The fundus image at the 48-month follow-up (D) showed extensive subretinal hemorrhage. ICGA images at the 48-month follow-up (E) showed an extensive area of hypofluorescence caused by masking. An OCT B-scan at the 48-month follow-up (F) showed subretinal fluid, subretinal hemorrhage and hemorrhagic PED.

**Additional file 5:** **Supplemental Figure 5. **Multimodal imaging of case 7 at baseline and 31-month follow-up. Fundus image at baseline (A) revealed pigmentary changes at the posterior pole. FFA at baseline revealed areas of hyperfluorescence (staining) at the macula. ICGA images (C) revealed areas of hypofluorescence (masking) at the macula. The fundus image at the 31-month follow-up (E) revealed a large PED at the macula. FFA at 31-month follow-up (F) revealed pooling hyperfluorescence corresponding to the PED adjacent to an intense localized area of hyperfluorescence. ICGA images (G and I) showed a hyperfluorescent “polyp” adjacent to an area of hypofluorescence (masking caused by PED). OCT B-scans (H and J) showed subretinal fluid, flat irregular PED and large serous PED.

**Additional file 6:** **Supplemental Table 1. **Treatment received and clinical features of all study eyes at the last follow-up. *MRT was defined as the maximum retinal thickness of the scanned area measuredon all OCT B-scans of the study eye. †Due to severe macular edema and extensive PED, the SFCT and MRT of case 3 as well as the SFCT of case 4 could not be measured. BCVA, best corrected visual acuity; LogMAR, logarithm of the minimum angle of resolution; CMT, central macular thickness; SFCT, subfoveal choroidal thickness; MRT, maximum retinal thickness; PDT, photodynamic therapy. 

## Data Availability

The data analyzed during the current study are not publicly available but are available from the corresponding author on reasonable request.

## Abbreviations

AT1: Aneurysmal type 1 neovascularization
BCVA: Best corrected visual acuity
CFH: Complement Factor H gene
CMT: Central macular thickness
CNV: Choroidal neovascularization
CSC: Central serous chorioretinopathy
CVH: Choroidal vascular hyperpermeability
EZ: Ellipsoid zone
FAF: Fundus autofluorescence
FCE: Focal choroidal excavation
FFA: Fundus fluorescence angiography
FIPED: Flat irregular pigment epithelium detachment
ICGA: Indocyanine green angiography
MRT: Maximum retinal thickness
PCV: Polypoidal choroidal vasculopathy
PDT: Photodynamic therapy
PED: Pigment epithelial detachment
PNV: Pachychoroid neovasculopathy
PPE: Pachychoroid pigment epitheliopathy
PPS: Peripapillary pachychoroid syndrome
RPE: Retinal pigment epithelium
SFCT: Subfoveal choroidal thickness
OCT: Optical coherence tomography
VEGF: Vascular endothelial growth factor

## References

[1] Warrow DJ, Hoang QV, Freund KB (2013). Pachychoroid pigment epitheliopathy. Retina.

[2] Cheung CMG, Lee WK, Koizumi H, Dansingani K, Lai TYY, Freund KB (2019). Pachychoroid disease. Eye (Lond).

[3] Karacorlu M, Ersoz MG, Arf S, Hocaoglu M, Sayman MI (2018). Long-term follow-up of pachychoroid pigment epitheliopathy and lesion characteristics. Graefes Arch Clin Exp Ophthalmol.

[4] Ersoz MG, Hocaoglu M, Sayman Muslubas I, Arf S, Karacorlu M (2021). Development of Pachychoroid Pigment Epitheliopathy and Transformation to Central Serous Chorioretinopathy after Intravitreal Dexamethasone Implantation. Retin Cases Brief Rep.

[5] Saito W, Hashimoto Y, Hirooka K, Ishida S (2021). Choroidal Thickness Changes in a Patient Diagnosed with Central Serous Chorioretinopathy during Follow-up for Pachychoroid Pigment Epitheliopathy. Retin Cases Brief Rep.

[6] Fung AT, Yannuzzi LA, Freund KB (2012). Type 1 (sub-retinal pigment epithelial) neovascularization in central serous chorioretinopathy masquerading as neovascular age-related macular degeneration. Retina.

[7] Siedlecki J, Schworm B, Priglinger SG (2019). The Pachychoroid Disease Spectrum-and the Need for a Uniform Classification System. Ophthalmol Retina.

[8] Ersoz MG, Arf S, Hocaoglu M, Sayman Muslubas I, Karacorlu M (2018). Indocyanine Green Angiography of Pachychoroid Pigment Epitheliopathy. Retina.

[9] Ersoz MG, Karacorlu M, Arf S, Hocaoglu M, Sayman MI (2018). Pachychoroid pigment epitheliopathy in fellow eyes of patients with unilateral central serous chorioretinopathy. Br J Ophthalmol.

[10] Pang CE, Freund KB (2015). Pachychoroid neovasculopathy. Retina.

[11] Dansingani KK, Balaratnasingam C, Klufas MA, Sarraf D, Freund KB (2015). Optical Coherence Tomography Angiography of Shallow Irregular Pigment Epithelial Detachments In Pachychoroid Spectrum Disease. Am J Ophthalmol.

[12] Cheung CMG, Lai TYY, Teo K, Ruamviboonsuk P, Chen SJ, Kim JE (2021). Polypoidal Choroidal Vasculopathy: Consensus Nomenclature and Non-Indocyanine Green Angiograph Diagnostic Criteria from the Asia-Pacific Ocular Imaging Society PCV Workgroup. Ophthalmology.

[13] Ersoz MG, Karacorlu M, Arf S, Hocaoglu M, Sayman MI (2018). Outer Nuclear Layer Thinning in Pachychoroid Pigment Epitheliopathy. Retina.

[14] Sakurada Y, Fragiotta S, Leong BCS, Parikh R, Hussnain SA, Freund KB (2020). Relationship between Choroidal Vascular Hyperpermeability, Choriocapillaris Flow Density, and Choroidal Thickness in Eyes with Pachychoroid Pigment Epitheliopathy. Retina.

[15] Castro-Navarro V, Behar-Cohen F, Chang W, Joussen AM, Lai TYY, Navarro R (2021). Pachychoroid: current concepts on clinical features and pathogenesis. Graefes Arch Clin Exp Ophthalmol.

[16] Kim K, Kim JM, Kim DG, Yu SY, Kim ES (2020). Five-Year Follow-Up of Unaffected Fellow Eyes in Patients with Polypoidal Choroidal Vasculopathy. Ophthalmologica.

[17] Baek J, Cheung CMG, Jeon S, Lee JH, Lee WK (2019). Polypoidal Choroidal Vasculopathy: Outer Retinal and Choroidal Changes and Neovascularization Development in the Fellow Eye. Invest Ophthalmol Vis Sci.

[18] Kang SW, Lee H, Bae K, Shin JY, Kim SJ, Kim JM (2017). Investigation of precursor lesions of polypoidal choroidal vasculopathy using contralateral eye findings. Graefes Arch Clin Exp Ophthalmol.

[19] Lee GW, Roh HC, Kang SW, Kim AY, Noh H, Choi KJ (2021). The implications of subretinal fluid in pachychoroid neovasculopathy. Sci Rep.

[20] Borooah S, Sim PY, Phatak S, Moraes G, Wu CY, Cheung CMG (2021). Pachychoroid spectrum disease. Acta Ophthalmol.

