# Supplementary figures and images for: Case series: pachychoroid pigment epitheliopathy transformed to polypoidal choroidal vasculopathy after long-term follow-up
Source: BMC Ophthalmol. 2022 Jun 21;22:272. doi: 10.1186/s12886-022-02487-8 (PMC9210595; doi:10.1186/s12886-022-02487-8)

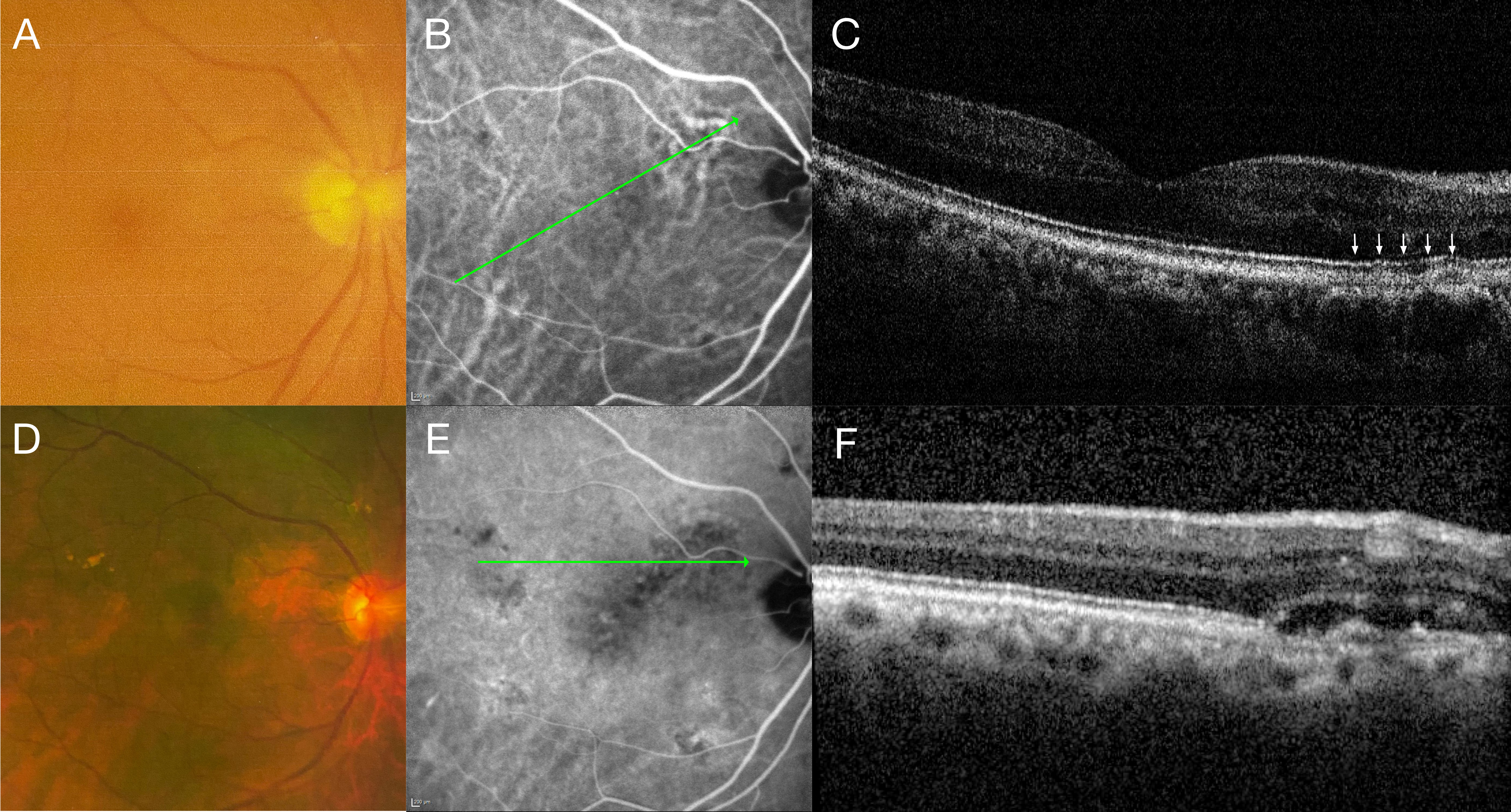

Supplement: Supplementary file 1 — Additional file 1: Supplemental Figure 1. Multimodal imaging of case 3 at baseline and 37-month follow-up. Fundus image at baseline (A) revealed pigmentary changes at the posterior pole. The ICGA image at baseline (B) revealed dilated choroidal vessels. An OCT-B scan at baseline (C) revealed flat irregular PED accompanied by pachyvessels corresponding to the location of pigmentary changes on fundus images. The fundus image at the 37-month follow-up (D) revealed subretinal fluid at the posterior pole. The ICGA image at the 37-month follow-up (E) revealed a hypofluorescent lesion (corresponding to the area with subretinal fluid) and hyperfluorescent “polyps”. An OCT B-scan at the 37-month follow-up (F) showed subretinal fluid and flat irregular PED. [file 12886_2022_2487_MOESM1_ESM.jpg]

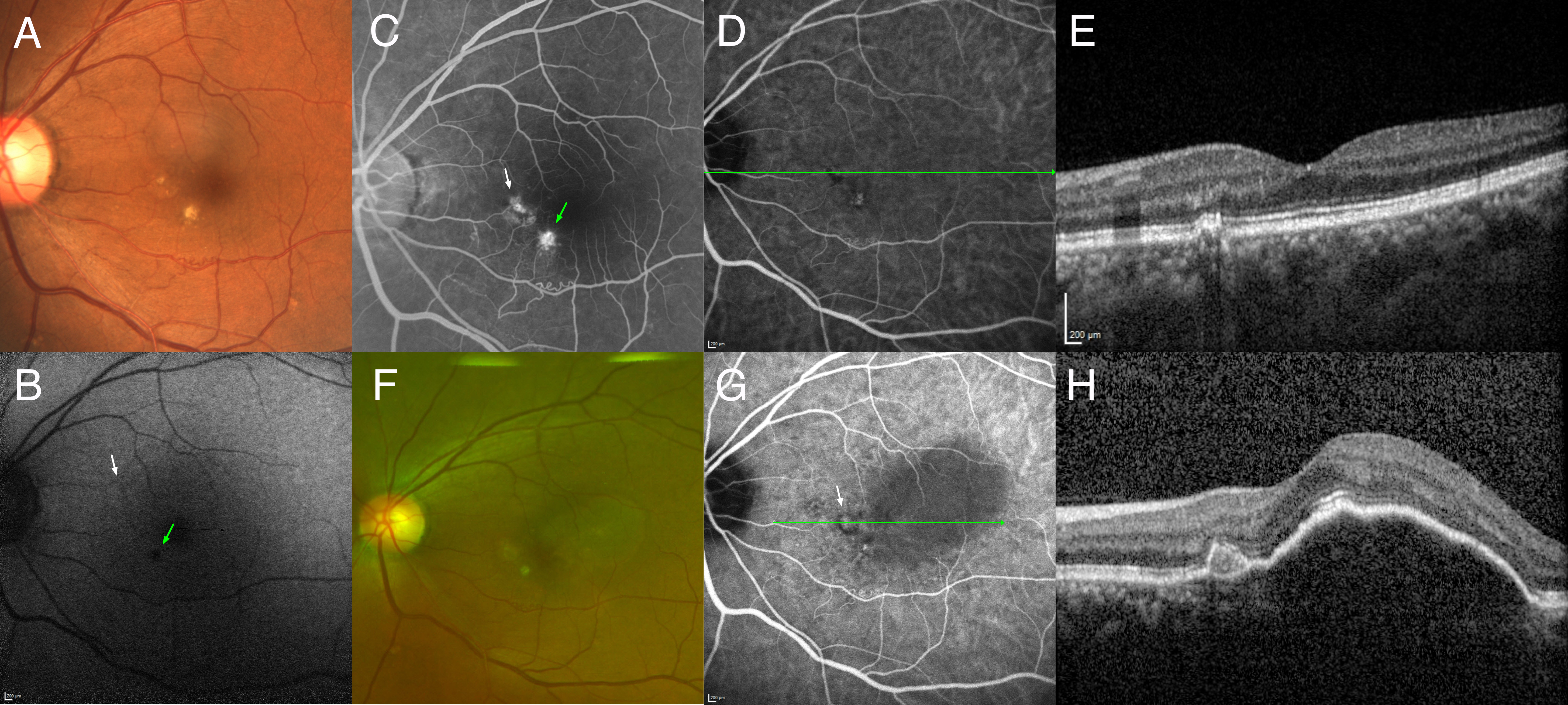

Supplement: Supplementary file 2 — Additional file 2: Supplemental Figure 2. Multimodal imaging of case 4 at baseline and 18-month follow-up. The fundus image at baseline (A) revealed two areas with pigmentary changes. Fundus autofluorescence (B) revealed a hyperautofluorescent lesion (indicated with white arrow) and a hypoautofluorescent lesion (indicated with green arrow). FFA at baseline (C) revealed two hyperfluorescent lesions; the white arrow indicates the area of staining hyperfluorescence, while the green arrow indicates the area of the window defect. (D) ICGA at baseline revealed a hyperfluorescent lesion corresponding to a PED on OCT B-scan (E). The fundus image at the 18-month follow-up (F) revealed extensive subretinal hemorrhage. ICGA images (G) at the 18-month follow-up revealed an area of hypofluorescence corresponding to the area with subretinal hemorrhage and a hyperfluorescent “polyp” (indicated with white arrow). [file 12886_2022_2487_MOESM2_ESM.jpg]

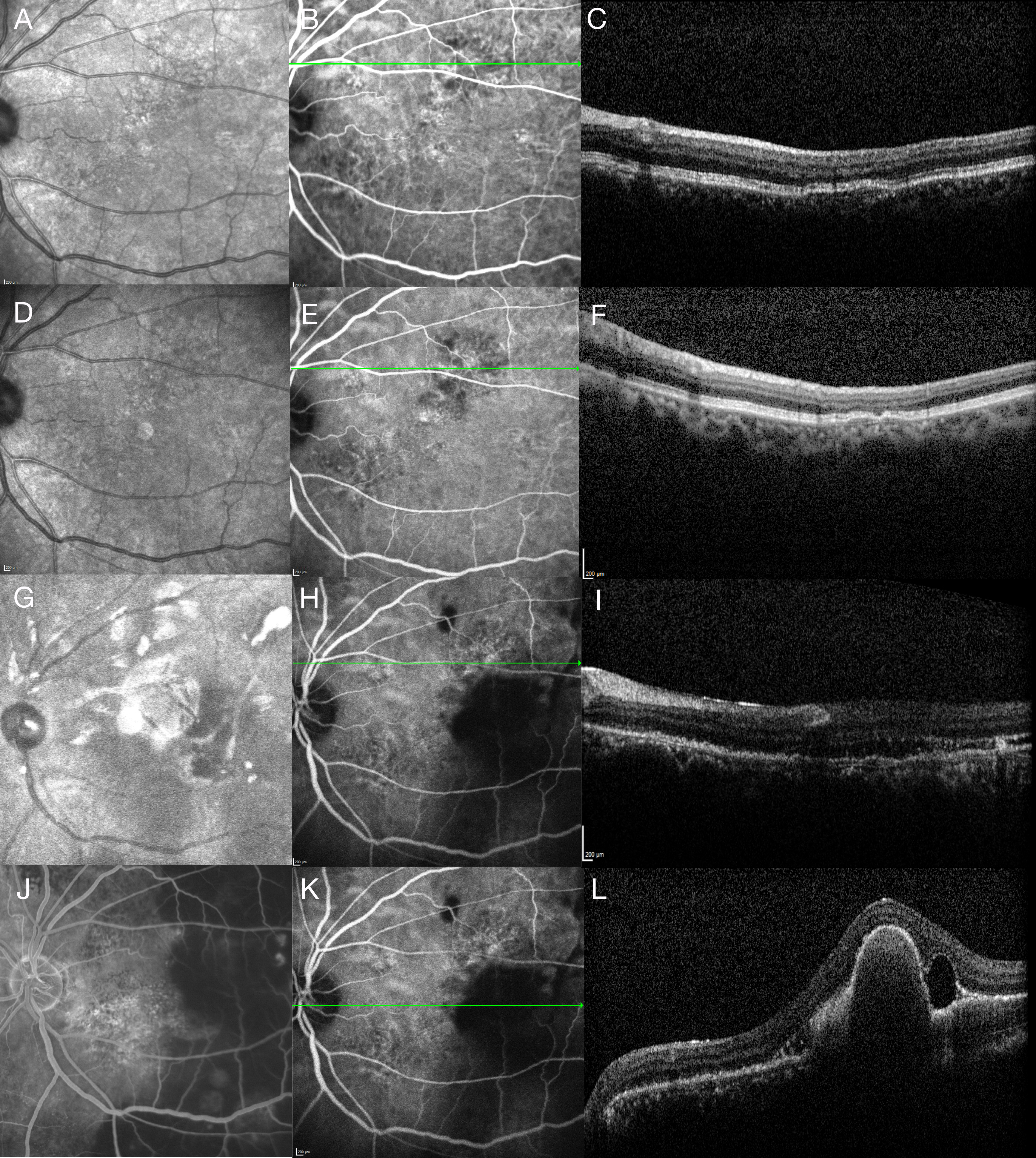

Supplement: Supplementary file 3 — Additional file 3: Supplemental Figure 3. Multimodal imaging of case 5 at baseline and follow-up. The infrared reflectance (IR) image at baseline (A) revealed mottled reflectivity at the posterior pole. ICGA images (B) revealed areas of hyper- and hypo-fluorescence corresponding to serous PED on OCT B-scan (C). IR image (D), ICGA image (E) and OCT B-scan at 61-month follow-up revealed a stable clinical course. The IR image at the 84-month follow-up (G) revealed a large area of decreased reflectivity corresponding to extensive subretinal hemorrhage. FA at the 84-month follow-up (J) revealed mottled hyperfluorescence adjacent to an extensive area of hypofluorescence (masking). ICGA images at the 84-month follow-up (H and K) revealed a branching vascular network adjacent to an extensive area of hypofluorescence caused by masking. An OCT B-scan revealed subretinal fluid with flat irregular PED (I) and hemorrhagic PED (L). [file 12886_2022_2487_MOESM3_ESM.jpg]

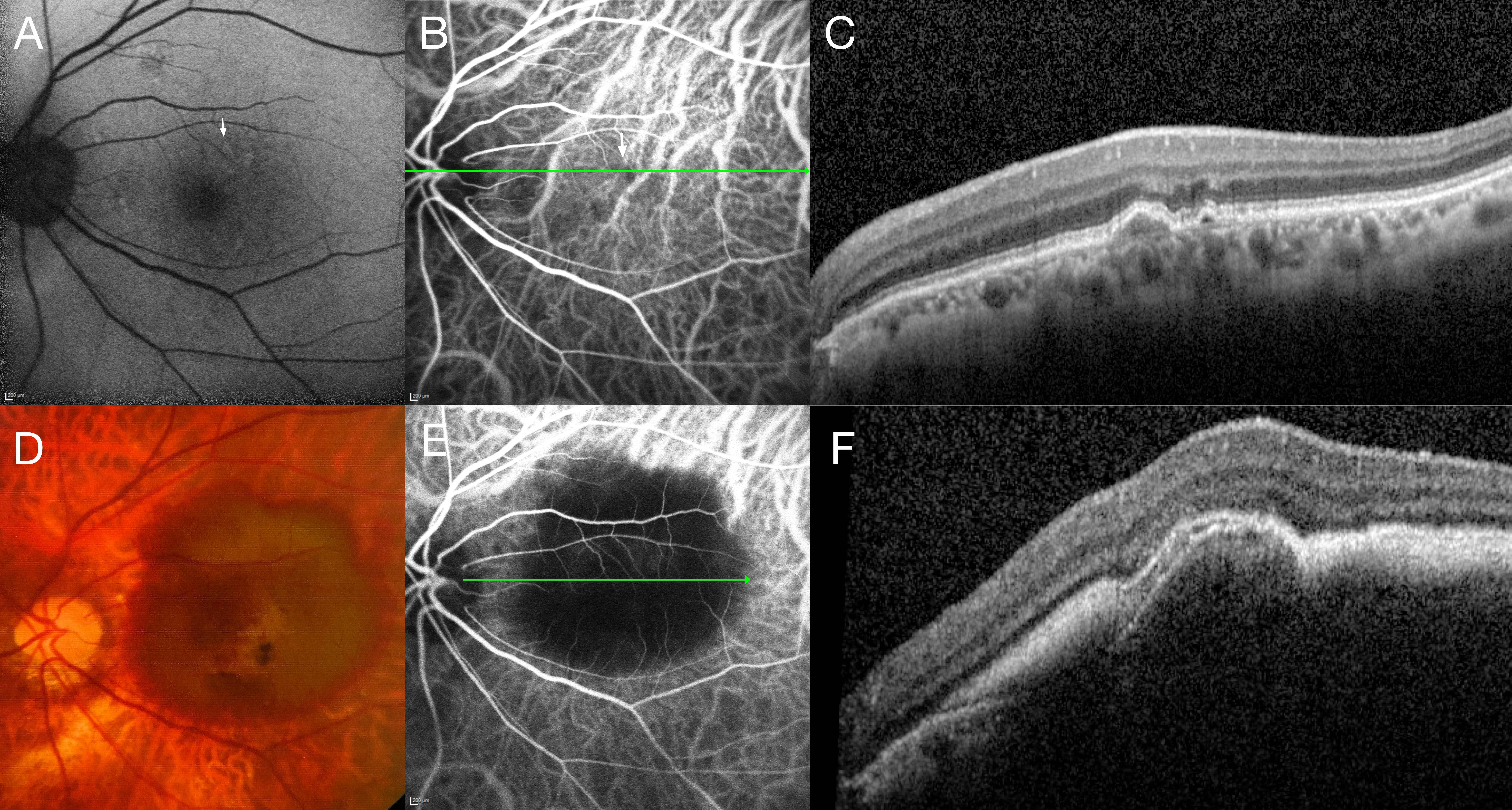

Supplement: Supplementary file 4 — Additional file 4: Supplemental Figure 4. Multimodal imaging of case 6 at baseline and 48-month follow-up. FAF at baseline (A) revealed an area of hyperautofluorescence corresponding to hypofluorescence (masking) on ICGA images (B). An OCT B-scan revealed irregular PED with hyperreflective material. The fundus image at the 48-month follow-up (D) showed extensive subretinal hemorrhage. ICGA images at the 48-month follow-up (E) showed an extensive area of hypofluorescence caused by masking. An OCT B-scan at the 48-month follow-up (F) showed subretinal fluid, subretinal hemorrhage and hemorrhagic PED. [file 12886_2022_2487_MOESM4_ESM.jpg]

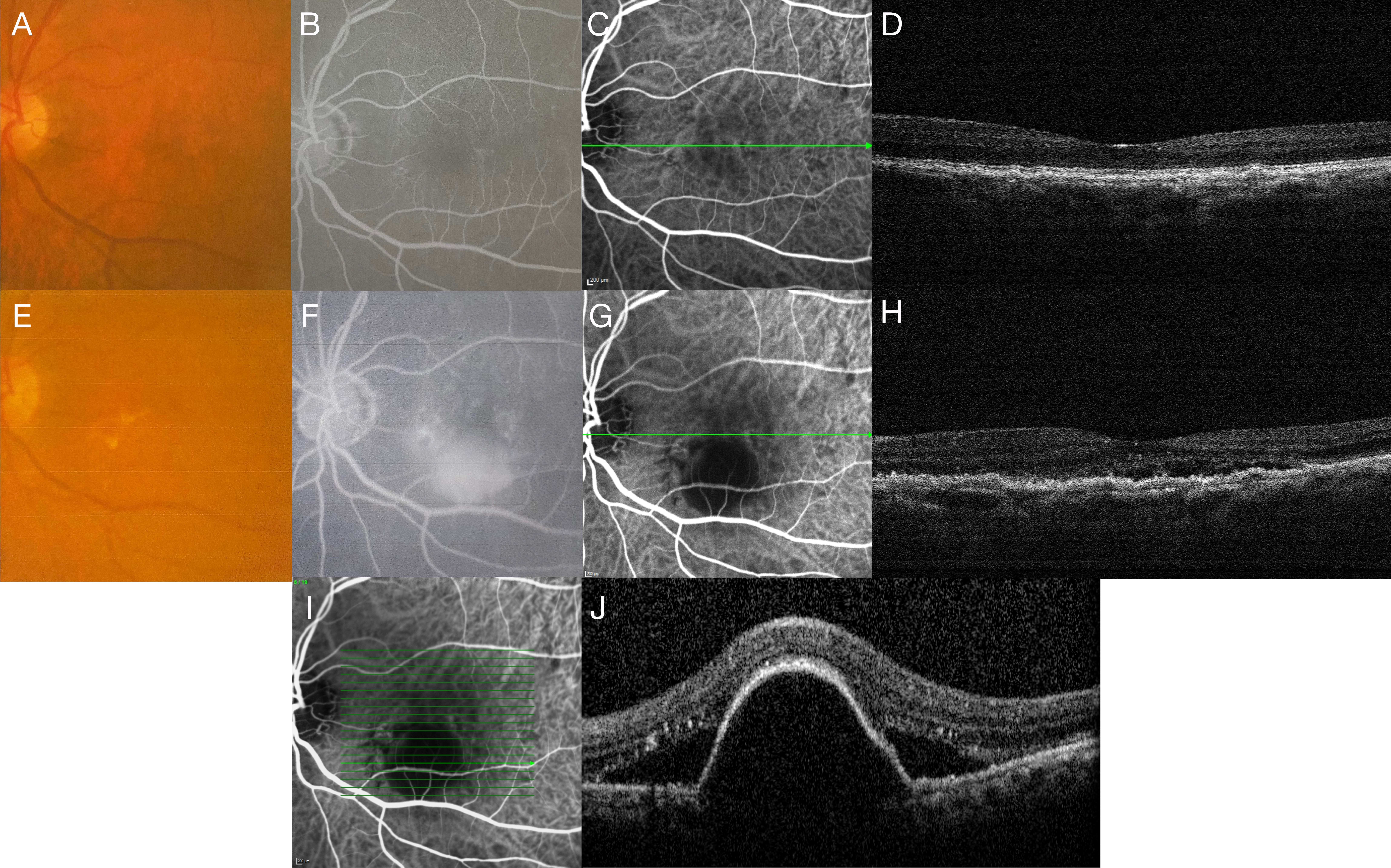

Supplement: Supplementary file 5 — Additional file 5: Supplemental Figure 5. Multimodal imaging of case 7 at baseline and 31-month follow-up. Fundus image at baseline (A) revealed pigmentary changes at the posterior pole. FFA at baseline revealed areas of hyperfluorescence (staining) at the macula. ICGA images (C) revealed areas of hypofluorescence (masking) at the macula. The fundus image at the 31-month follow-up (E) revealed a large PED at the macula. FFA at 31-month follow-up (F) revealed pooling hyperfluorescence corresponding to the PED adjacent to an intense localized area of hyperfluorescence. ICGA images (G and I) showed a hyperfluorescent “polyp” adjacent to an area of hypofluorescence (masking caused by PED). OCT B-scans (H and J) showed subretinal fluid, flat irregular PED and large serous PED. [file 12886_2022_2487_MOESM5_ESM.jpg]
