# Supplementary material for: Case series: pachychoroid pigment epitheliopathy transformed to polypoidal choroidal vasculopathy after long-term follow-up
Source: BMC Ophthalmol. 2022 Jun 21;22:272. doi: 10.1186/s12886-022-02487-8 (PMC9210595; doi:10.1186/s12886-022-02487-8)
Supplement: Supplementary file 6 — Additional file 6: Supplemental Table 1. Treatment received and clinical features of all study eyes at the last follow-up. *MRT was defined as the maximum retinal thickness of the scanned area measuredon all OCT B-scans of the study eye. †Due to severe macular edema and extensive PED, the SFCT and MRT of case 3 as well as the SFCT of case 4 could not be measured. BCVA, best corrected visual acuity; LogMAR, logarithm of the minimum angle of resolution; CMT, central macular thickness; SFCT, subfoveal choroidal thickness; MRT, maximum retinal thickness; PDT, photodynamic therapy. [file 12886_2022_2487_MOESM6_ESM.docx]

Supplemental Table 1. Treatment received and clinical features of all study eyes on the last follow-ups.

| Patient, n | BCVA  Snellen/logMAR | CMT  (µm) | SFCT  (µm) | MRT*  (µm) | No. of anti-VEGF injections | No. of laser session | No. of PDT session | No. of vitrectomy surgery |
| --- | --- | --- | --- | --- | --- | --- | --- | --- |
| 1 | 0.10/1.00 | 285 | 121 | 659 | 29 | 0 | 1 | 0 |
| 2 | 0.12/0.90 | 173 | 226 | 300 | 6 | 0 | 1 | 0 |
| 3† | 0.02/1.70 | 2099 | N/A | N/A | 14 | 0 | 1 | 1 |
| 4† | 0.05/1.30 | 327 | N/A | 872 | 8 | 1 | 0 | 0 |
| 5 | 0.04/1.40 | 372 | 401 | 614 | 1 | 0 | 0 | 1 |
| 6 | 0.20/0.70 | 209 | 191 | 430 | 8 | 0 | 1 | 0 |
| 7 | 0.20/0.70 | 240 | 195 | 572 | 2 | 0 | 1 | 0 |

* MRT was defined as the maximum retinal thickness of the scanned area measured on all OCT B-scans of the study eye.

†Due to severe macular edema and extensive PED, the SFCT and MRT of case 3 as well as the SFCT of case 4 could not be measured.

BCVA, best corrected visual acuity; LogMAR, logarithm of the minimum angle of resolution; CMT, central macular thickness; SFCT, subfoveal choroidal thickness; MRT, maximum retinal thickness; PDT, photodynamic therapy.
